# Cytotoxic Evaluation and Determination of Organic and Inorganic Eluates from Restorative Materials

**DOI:** 10.3390/molecules26164912

**Published:** 2021-08-13

**Authors:** Konstantina Roussou, Alexandros K. Nikolaidis, Fani Ziouti, Aristidis Arhakis, Konstantinos Arapostathis, Elisabeth A. Koulaouzidou

**Affiliations:** 1 Department of Pediatric Dentistry, School of Dentistry, Aristotle University of Thessaloniki, 541 24 Thessaloniki, Greece; roussaki90@gmail.com (K.R.); arhakis@dent.auth.gr (A.A.); koarap@dent.auth.gr (K.A.); 2 Division of Dental Tissues’ Pathology and Therapeutics (Basic Dental Sciences, Endodontology and Operative Dentistry), School of Dentistry, Aristotle University of Thessaloniki, 541 24 Thessaloniki, Greece; fziouti@gmail.com (F.Z.); koulaouz@dent.auth.gr (E.A.K.)

**Keywords:** composite resins, gas chromatography, ion release, cytotoxicity

## Abstract

Over the last years, diverse commercial resin-based composites have dominated as dental filling materials. The purpose of the present study was to determine organic and inorganic eluates from five restorative materials using GC/MS and ICP–OES and to compare the effect on cell survival of human gingival fibroblasts of a conventional and a bioactive resin. Five commercially available restorative materials were employed for this study: Activa^TM^ Bioactive Restorative, ENA HRi, Enamel plus HRi Biofunction, Fuji II LC Capsule, and Fuji IX Capsule. Disks that were polymerized with a curing LED light or left to set were immersed in: 1 mL methanol or artificial saliva for GC/MS analysis, 5mL deionized water for ICP–OES, and 5mL of culture medium for cell viability. Cell viability was investigated with a modified staining sulforhodamine B assay.The following organic substances were detected: ACP, BHT, BPA, 1,4-BDDMA, CQ, DBP, DMABEE, HEMA, MCE, MeHQ, MOPA, MS, TMPTMA, and TPSb and the ions silicon, aluminum, calcium, sodium, and barium. Activa Bioactive Restorative was found to be biocompatible. Elution of organic substances depended on material’s composition, the nature of the solvent and the storage time. Ions’ release depended on material’s composition and storage time. The newly introduced bioactive restorative was found to be more biocompatible.

## 1. Introduction

In the last decades resin-based composites are the most commonly used dental filling materials and have been proposed as an alternative solution to amalgam, gold and ceramic restorations. Resin-based materials consist of an organic matrix which is reinforced with inorganic fillers and silane coupling agents that connect the resin matrix with the fillers [1]. Usually, the resin matrix consists of base monomers bisphenol A-glycidyl methacrylate and/or urethane dimethacrylate (e.g., BisGMA and/or UDMA), co-monomers (e.g., triethyleneglycol dimethacrylate-TEGDMA and ethylene glycol dimethacrylate-EGDMA), and other additives such as photoiniators (e.g., camphoroquinone) and polymerization inhibitors (e.g., BHT). During the polymerization process of the resin material, the base monomers and the comonomers form a three dimensional network structure [2,3]. Complete polymerization is not possible because the crosslinking process may be controlled by diffusion phenomena domaining inside the network [4]. The ingredients that are not bonded to the network may be released in the oral cavity [4]. Except for the elution of residuals monomers, which usually happens immediately after placement of the dental restorative material, other physical and chemical processes such as wear, erosion, hydrolysis and solvolysis may promote a constant degradation of the material [5]. Numerous studies have shown release of components from resin-based materials in different solvents, while there are several factors that affect the substances’ release such as the extend of polymerization and network’s complexity, the matrix’s composition [6], the properties of the elutable substances such as hydrophilicity, chemical composition, molecular weight [7,8], the porosity of the material and specimen’s thickness [6].

Public concerns have been raised about adverse biological effects due to the release of these ingredients into the oral cavity [5], since some of these ingredients have exhibited cytotoxic [9,10,11,12,13], genotoxic effects [12,13] or cause allergic reactions [14]. What is more, some of them such as Bisphenol A (BPA)—usually associated with BisGMA production—demonstrate estrogenic activity [15]. The elution of these components influences both the structural stability and the biocompatibility of the material, which is of great importance. These components may be released into salivary fluids and contact the mucosal tissues or diffuse through dentine towards the pulp [6]. Several analytical procedures and different organic solvents that simulate oral conditions have been proposed in literature in order to identify the eluted substances from resin-based materials [4,7,16,17,18]. All methods seem to be sufficient for identification of the eluted monomers, but there are differences among them, as far as the molecular weight of the eluted substances, the identification of by-products and degradation products and the quantification of the substances concerns [18]. High-performance liquid chromatography (HPLC) or HPLC/mass spectrometry (HPLC/MS) is commonly used to identify large molecular size monomers, such as base monomers such as BisGMA, UDMA, and BisEMA [19,20,21], while gas chromatography/mass spectrometry (GC/MS) is more suitable to identify additives, smaller monomers, comonomers and other volatile compounds due to base monomers’ decomposition and fragmentation products [18,22]. 

On the other hand, efforts have been made in order to develop materials that combine characteristics of composites resins with glass ionomers [23,24]. These materials would promote remineralization of dental tissues and offer antimicrobial activity to reduce the risk of developing secondary caries due to ions’ release [23,24,25,26]. There are two groups of resin-based materials capable of releasing ions, the ones that contain modified/functionalized calcium phosphate particles and the ones that contain ion-releasing fillers with a reinforcing phase [24]. The dispersed phase (e.g., inorganic fillers) determines the mechanical and physical characteristics of resin composites [27]. Fillers can influence fracture resistance, polymerization shrinkage and thermal expansion [27]. Surface prereacted glass ionomer (S-PRG) fillers are produced by an acid-based reaction between polyacrylic acid solution and fluro-boro-aluminosilicate glass [28] and are capable of releasing ions such as aluminum, boron, fluoride, sodium, silicon and strontium [29]. 

Recently, bioactive glass has been added into dental materials in order to promote remineralization and hard tissue formation [30,31], and a series of products that according to manufacturer’s contain bioactive glass filler has been introduced in the dental market under the trade name Activa^TM^ Bioactive (Pulpdent, Watertown, MA, USA). These materials contain patented bioactive ionic and moisture friendly resin, patented rubberized resin and reactive glass fillers, while they are known as bisphenol A and bisphenol A glycol dimethacrylate (Bis-GMA) free. Three chemical reactions take place during their setting process: chemical curing, light polymerization and acid-base reaction [30,32,33]. The newly introduced bioactive materials are supposed to release and recharge with calcium, phosphate and fluoride ions during the pH cycles of ionic exchange between teeth and saliva [30]. 

Cell populations that simulate oral conditions have been used in order to study the cytotoxicity of dental materials [34]. Usually gingival fibroblast, keratinocytes of the oral epithelium and standardized strains of mouse L-929 or 3T3 fibroblasts are used for cytotoxicity studying [18]. The cytotoxic effects of the eluted substances have been studied by tests that estimate the severity of apoptotic action, the damage to ribonucleic acid and glutathione level in cells [34].

Taking into consideration the above information mentioned, it becomes clear that resin-based materials are complex materials in terms of their chemical composition and newly introduced materials become available in everyday clinical practice. Therefore, the aim of this study was to determine organic and inorganic species potentially eluted from five restorative materials by the combined use of gas chromatography-mass spectrometry (GC/MS) and inductively coupled plasma–optical emission spectrometry (ICP–OES) techniques, respectively, and to estimate the possible biological effect of a “conventional” resin composite and the newly introduced bioactive resin on cell survival of human gingival fibroblasts. The null hypothesis was that no organic and inorganic eluates would be released from the restorative materials and that neither the newly introduced bioactive resin-based restorative material nor the “conventional” resin-based restorative material would influence cells’ viability. 

## 2. Results

### 2.1. Organic Eluates Determination in Methanol and Artificial Saliva by Using GC/MS

**Figure 1:**
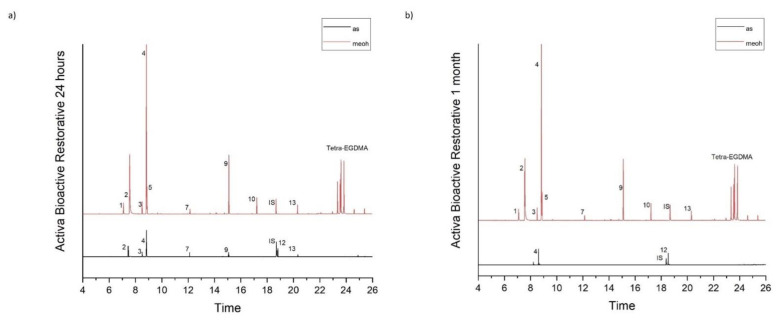
(**a**,**b**). Chromatograms of Activa Bioactive Restorative in artificial saliva and methanol for both observation periods (24 h and 1 month).

**Figure 2:**
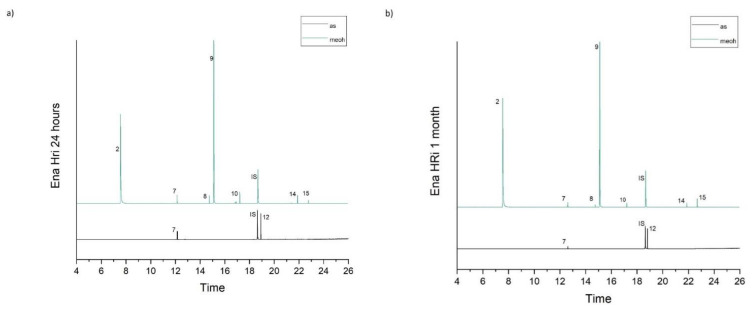
(**a**,**b**). Chromatograms of Ena HRi in artificial saliva and methanol for both observation periods (24 h and 1 month).

**Figure 3:**
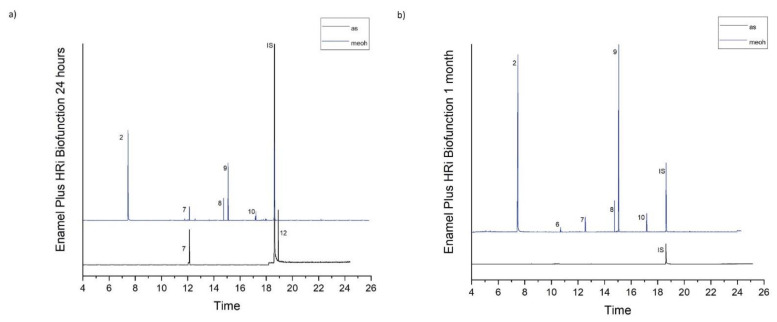
(**a**,**b**). Chromatograms of Enamel Plus HRi Biofunction in artificial saliva and methanol for both observation periods (24 h and 1 month).

**Figure 4:**
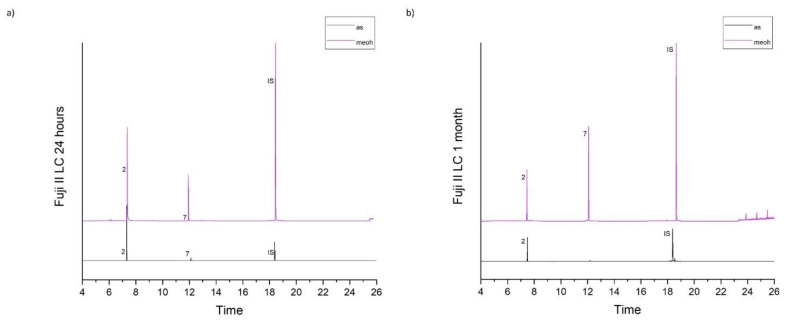
(**a**,**b**). CChromatograms of Fuji II LC in artificial saliva and methanol for both observation periods (24 h and 1 month).

**Figure 5:**
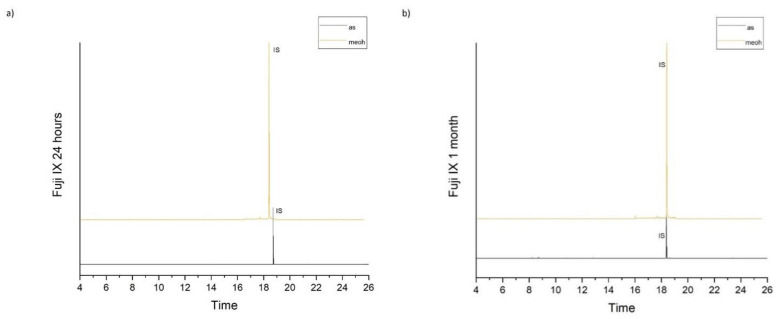
(**a**,**b**). Chromatograms of Fuji IX in artificial saliva and methanol for both observation periods (24 h and 1 month).

Table 1 accumulates the GC-MS identification data for the total compounds eluted from the examined dental materials. The analytes detected in the methanolic and artificial saliva eluents for each material are summarized in Table 2 and Table 3, while the median values and interquartile range of the eluted substances are listed in Table 4 and Table 5. Representative chromatograms of each material in both solvents and for both observation periods are depicted in Figure 1, Figure 2, Figure 3, Figure 4 and Figure 5.

It is shown that no substance was detected from the glass ionomer cement Fuji IX fast, while the following substances were detected in artificial saliva extracts: Acetophenone (ACP), 1,4-butylene glycol dimethacrylate (1,4-BDDMA), Camphorquinone (CQ), Dibutyl phthalate (DBP), 2-Hydroxyethyl methacrylate (HEMA), Methoxyphenyl acetic acid (MOPA), and Trimethylolpropane trimethacrylate (TMPTMA). CQ was present in all materials at 1 day observation period, with Fuji II LC presenting the lowest concentration and Ena HRi the highest concentration, HEMA was detected in Activa Bioactive Restorative and Fuji II LC, while DBP was detected in Activa Bioactive Restorative, Ena HRi and Ena HRi Biofunction, and finally ACP, 1.4-BDDMA, MOPA and TMPTMA were found only in Activa Bioactive Restorative artificial saliva eluates. Ena HRi presented significantly higher release of CQ and DBP.

More substances were detected in methanol extracts from the resin-based materials. Specifically, the following substances were detected: α-Methylstyrene (MS), 2-Hydroxyethyl methacrylate (HEMA), Acetophenone (ACP), Butylated hydroxytoluene (BHT), Bisphenol A (BPA), 1,4-butylene glycol dimethacrylate (1,4-BDDMA), Camphorquinone (CQ), Ethyl-4-(dimethylamino)benzoate (DMABEE), Methoxyphenyl acetic acid (MOPA), Methyl cumyl ether (MCE), Mequinol (MeHQ), Trimethylolpropanetrimethacrylate (TMPTMA) and Triphenylstibine (TPSb). The majority of the substances presented an increase in their concentration in methanolic extracts at one month observation period. Activa Bioactive restorative presented significantly higher release of HEMA, CQ and DMABEE from all the other materials for both observation periods, while Ena HRi presented significant higher elution of 1,4-BDDMA when compared to all other materials for both study periods. MS, ACP, MCE, MOPA and TMPTMA were released only from Activa Bioactive Restorative for both observation periods.

### 2.2. Ions Release in Deionized Water by Using ICP–OES

The ions silicon, aluminum, calcium, sodium, and barium were detected in all materials’ eluates, while phosphorus was not detected in any of the tested extract. Generally, the ions release decreased over time. Fuji IX had the highest release of silicon, aluminum, and sodium for both observation periods. Release of calcium was increased at two weeks observation period for all materials apart from Fuji II LC. Mean values (are given in ppm) and standard deviations of the released ions are listed in Table 6.

### 2.3. Cell Viability

**Figure 6:**
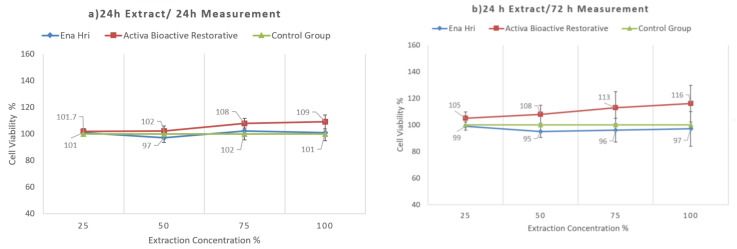
The viability of cells exposed to 24 h extracts of Activa Bioactive Restorative and Ena HRi at (**a**) 24 h and (**b**) 72 h.

**Figure 7:**
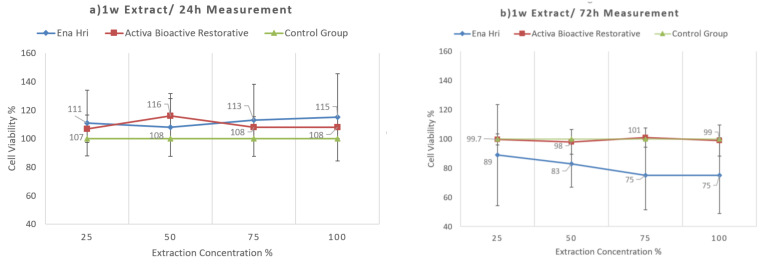
The viability of cells exposed to 1 week extracts of Activa Bioactive Restorative and Ena HRi at (**a**) 24 h and (**b**) 72 h.

Activa Bioactive Restorative seems to be biocompatible. Time of extraction was not found to be critical, but time of exposure was found to be critical, since in both extraction times and 72 h exposure Ena HRi was more cytotoxic than Activa Bioactive Restorative. At 24 h, exposure for both extraction times both materials exhibited a similar cytocompatibility profile with no statistically significant differences (Figure 6 and Figure 7). Table 7 shows the mean values and standard deviations for both time of extraction and both time of exposure.

## 3. Discussion

Dental composites are made up from monomers that are polymerized to form a polymer matrix, (organic) organically modified inorganic fillers and/or inorganic reinforcing fillers, chemical substances that either initiate and promote or inhibit the polymerization reaction and coupling agents that help fillers’ adhesion to the matrix [6]. Previous studies have shown that anything present in dental composites may be eluted into proper solvents [4,5,16,22,35,36]. In the present study, several eluted monomers were detected after storage of the five materials in methanol and artificial saliva solutions. In methanol solutions, more substances were detected and in higher concentrations than in artificial saliva. These findings are in accordance with previous studies that also presented more substances to be found in methanolic or ethanolic extracts than in artificial or native saliva or water extracts [4,16,22,37,38]. An increased elution was expected, since organic solvents (such as methanol) are able to penetrate easier and deeper into the organic matrix than other solvents such as water or artificial saliva [18]. This penetration of methanol molecules not only causes divergence of the polymer chains leading to the swelling of the polymer network and diffusion of the unreacted monomers but also softening of the matrix and further penetration of the solvent [7,39,40]. Ethanol 75% is described by the Food and Drug Administration (FDA) as “*food simulation and aging accelerator*” and is proved that methanol and ethanol 75% have similar dissolving capacity [5]. According to the above, organic solvents are capable to simulate degradation and erosion of the resin-based materials [18]. Therefore, in this study, artificial saliva was used in order to simulate oral conditions and methanol to simulate extreme oral conditions, artificial aging and achieve the highest possible elution of monomers. 

However, not all substances followed the increased elution in methanolic solutions. Specifically, DBP, which belongs to phthalates and is used as a plasticizer that impart flexibility and durability [41], was only found in artificial saliva solutions of Ena HRi in significantly higher concentrations than Activa Bioactive Restorative and Enamel Plus Biofunction and not in methanol. This finding comes in contrast with previous studies suggesting that only ethyl alcohol diffuse phthalates esters and elution in water at 37 °C is not likely to occur [42,43]. This elution in artificial saliva could be explained by the fact that DBP is more soluble in aqueous solutions than in methanol solutions [44,45]. Commonly used plasticizers are relatively considered nontoxic, but there are studies that support a synergic cytotoxic action when phthalates are combined with other organic monomers [46,47], while there are studies that reveal hormonal activity derived from phthalates esters [48,49,50]. 

Activa Bioactive Restorative is declared to be a bioactive ionic resin with reactive glass fillers, particularly, a blend of diurethane and other methacrylates with modified polyacrylic acid containing amorphous silica and sodium fluoride. In this material, MOPA was found to be the dominant compound among the substances eluted, and its concentration was significantly higher at one month observation period in methanolic solutions. MOPA was eluted only from Activa Bioactive Restorative and, along with its derivatives, has fungicidal activity against specific fungal strains [51,52]. Additionally, during the analysis, this material presented a complex initiation system, which could possibly relate to the three chemical reactions taking place during the setting process. Acetophenone (ACP) and its derivatives (such as 2,2-dimethoxy-2-phenyl acetophenone (DMPA)) are used as type I photoinitiators giving free radicals due to a unimolecular bond cleavage [53,54,55,56,57]. Traces of methylstyrene (MS) could associate with methylstyrene dimer which serves as a reversible addition-fragmentation chain-transfer (RAFT) agent in the synthesis of branched nanogels, containing UDMA crosslinkers, probably used as shrinkage and stress-limiting resin additives [58]. When taking into consideration the high-temperature conditions during the analytical process of gas chromatography, ACP, MS and methyl cumyl ether (MCE) seem to be potential thermal degradation products of cumyl hydroperoxide or dicumyl peroxide [59]. These are usually used in redox initiator systems promoting the free-radical polymerizations of methacrylate monomers at low temperatures [60]. What is more, DMABEE, which is a well-known tertiary amine contributing to such a redox initiation system, was also eluted from this material [61]. DMABEE has been found to exhibit cytotoxic effects and accumulation or integrity disruption of the cell membrane [5]. Finally, one more photoiniator, camphorquinone (CQ) was detected. DMABEE and CQ are commonly used in conjunction [16]. Previous studies have reported that DMABEE can be easily attached to the polymer network, though CQ may be free to elute from the matrix [62]. Consequently, it is expected of CQ to elute in higher concentrations than DMABEE [62]. By contrast, in the present study, DMABEE was eluted in higher concentrations than CQ from Activa Bioactive Restorative, Ena HRi and Enamel Plus Biofunction, while only CQ was found in Fuji II LC methanol extracts. These findings are in accordance with results published by Michelsen et al. [16]. The steric bulk of chiral structure of camphorquinone may lead to decreased diffusion rate to methanol despite the relatively low molecular weight, thus explaining the lower elution of CQ compared to DMABEE [30]. CQ may exhibit cytotoxic effects or cause cycle arrest and apoptosis of pulp cells [63]. Activa Bioactive Restorative was found to elute significantly more DMABEE than the other materials in methanol solutions for both observation periods.

Although HEMA was declared only in Fuji II LC MSDS, HEMA was eluted from all resin-based materials in methanol solutions. HEMA participates to the resin-modified polyacid chain formation and functions as a comonomer in the crosslinking process [64]. HEMA was found in significantly higher concentrations in Activa Bioactive Restorative methanolic solutions at both observation periods. HEMA eluted from Fuji II LC was also reported in previous studies [4,16,65]. Since HEMA elution has been connected with thermal fragmentation of UDMA during gas chromatography analysis [22], it could be an indirect evidence of unreacted UDMA monomer [66]. HEMA is found to be highly hydrophilic which in combination with its low molecular weight leads to diffusion through dentin, reaching the pulp and provoke adverse pulpal reaction [67]. 1,4-Butanediol dimethacrylate is another monomer usually used with main methyl methacrylates [68]. Ena HRi presented higher elution of 1,4-BDDMA in methanol solutions for both periods studied. 1,4-BDDMA has been found to induce cell differentiation at toxic concentrations but not to promote increase inROS production in HL-60 cells [69]. A third comonomer trimethylolpropane trimethacrylate (TMPTMA) was only eluted from Activa Bioactive Restorative. It is a trifunctional methacrylate monomer usually used in dental resins in order to improve flexural strength, hardness, absorption, and crosslink density [70] and wear properties while functioning as a crosslinking agent in the polymer matrix [71].

BPA is an organic monomer usually found in food contact materials such as drinking bottles and cups or food containers [72]. Nowadays, public concerns have been raised due to leaching of BPA from several products, since it has been linked to provoke a number of adverse effects including carcinogenicity, mutagenicity, estrogenic and apoptosis inducing activity [73,74,75]. BPA is characterized as a xenoestrogen and endocrine disruptor because it presents similar estrogenic activity to normal estrogens, when connected to the human estrogenic receptors [18]. In our study, traces of BPA were detected only in methanol extracts from Ena HRi. In dental materials, BPA is employed for the synthesis process of main matrix monomers such as dimethacrylate monomers [76]. During the curing process, BPA may not link to the polymer network and remain inside the material. As a result, BPA could easily leach when the material is immersed in organic solvents or water [77]. Taking into consideration the above, elution of BPA could either result from impurity of Bis-GMA or as degradation product of Bis-GMA and Bis-DMA [78,79]. From the tested materials in this study, Bis-GMA was a main component only in Ena HRi according to MSDS, which is the material that BPA was eluted from. Detection of BPA is a controversial issue among studies, since a very precise with low quantification limits method is needed to identify BPA [18]. Additionally, previous studies stated that detection of TEGDMA could be misinterpreted as detection of BPA [2,80]. This error is less possible to occur when using GC/MS, since both retention time and the mass spectra of these two substances differ greatly [76].

Polymerization inhibitors are added to dental materials to prevent polymerization during storage and application. BHT is a commonly used inhibitor and was eluted from Ena HRi and Enamel Plus Biofunction in methanol extracts in our study, with significantly higher elution from Enamel Plus Biofunction for both observation periods. BHT has been found to provoke allergic reactions [81] and hepatic cell toxicity [5]. What is more, a second inhibitor, MeHQ was found in Enamel Plus Biofunction methanolic extracts at one month observation period. MeHQ is a hydrophilic substance, while BHT is hydrophobic, and perhaps this difference in hydrophilicity is the reason why two inhibitors are added in dental materials [76]. 

A possible way to promote tooth remineralization reduce development of secondary caries, remineralize the hybrid layer, prevent enzymatic and hydrolytic degradation of the bonded interface has been the incorporation of calcium orthophosphates and bioactive glass particles in resin-based materials [82,83,84,85,86,87,88]. In this way, the materials behave as ions source, which would release calcium and phosphate to the liquid medium and thus create a supersaturated environment, which in turn would favor mineral deposition on the restoration/tooth interface [87]. Dental resin materials stored in artificial saliva release filler elements quicker than those stored in distilled water [89]. The fillers leach into distilled water due to stress corrosion of the glass and siloxane bonds are broken because of the silica’s hydrolysis [89].

The composition of the materials is given according to the Material Safety Data Sheets. These documents are created in accordance with the current legislation in order for the material to obtain the approval needed to be placed on the market. As a result, perhaps inorganic bioactive fillers (e.g., fluoroaluminate silicate glass, amorphous calcium phosphate, calcium chloride), according to previous studies ingredients, are not listed in the MSDS if the manufacturer was not obliged to declare every component of the material, always taking into account the maintenance of the patent’s “know how” [90,91]. As far as the detection of Ba concerns, detection of Ba derives from barium sulfate, which is usually added in the dental resin-based materials in order to act as radiopaque agent. What is more, according to the manufacturer statement: ACTIVA BioACTIVE plays an active role in maintaining oral health with release and recharge of significant amounts of calcium, phosphate and fluoride. These mineral components stimulate formation of a protective/connective apatite layer and a natural bonded-seal at the material-tooth interface. ACTIVA BioACTIVE’s ionic resin contains phosphate acid groups that improve the interaction between the resin and the reactive glass fillers and enhance the interaction with tooth structure. Through an ionization process that is dependent upon water, hydrogen ions break off from the phosphate groups and are replaced by calcium in tooth structure. This was the main reason that we examined a possible phosphorous release. Given that bioactive ionic resin is capable of providing phosphorous, a long-life recharging mechanism of phosphorous may demand not only the contribution of the surface available ionic resin but also the bulky components. Under the aforementioned consideration, we focused on the monitoring of a total phosphorous release. Furthermore, in the present study, a common approach to detect both organic and inorganic release was selected on the basis of overall diffusion phenomena not only limited from the material’s surface but also extended from the bulk to the simulated oral environment. In this context, the monitoring of the P ion mobility/release could be more effective by analyzing water leachates of dental materials with ICP–OES.

Ions’ release behavior varied upon the material type and the ions detected in the present study were expected from materials containing reactive glass [92]. The ions Si, Al, Ca, Na and Ba were released from all materials, but P was not detected. In general, ions release presented a decrease in their concentrations over time which is in accordance with previous studies [93,94]. Additionally, our results are in accordance with a recent study reporting release of Si, Al, Ca and Na from Activa Bioactive [95]. Phosphorus is biologically beneficial since it is a key component of teeth and bones in the form of phosphate [92]. Due to its high affinity for calcium, they form a variety of calcium phosphates with the most important being hydroxyapatite, additionally no health hazards are connected with phosphate [92]. In a previous study, a similarity in the release profile of phosphorus and aluminum has been reported, and an association of these two elements has been suggested [94]. However, data obtained from this study and previous studies imply that there is not a comparable association [93].

Aluminum forms complex ions with fluoride, such as AlF^2+^ and AlF_2_^+^, in aqueous solutions and decrease the level of free fluoride [92]. Although there have been some concerns about the effect of aluminum on health [96], it is stated that water consumption with 1000 ppm aluminum did not have adverse effects [97]. Silicon is also considered not to present any health hazards [92]. Usually, silicon is released in oxygenated forms such as silicate, which is also of low toxicity and appears to be beneficial for the circulatory system as it lows blood cholesterol levels [98]. When silicon is present as silicic acid, Si(OH)_4_, it reacts with aluminum to form hydroxyaluminosilicate complexes that are condensed to form a polymeric structure. As a result, any potential harmful species are removed from aqueous solutions, and the biological hazard of aluminum is eliminated [99]. Aluminum from glass filler particles replaces in part the silicon to form Si-O-Al bonds. These silicon substitutions with aluminum results in a basic pH and creation of negative sites [100]. Negative sites render glass particles to be more susceptible to acid attack. Sodium is also attracted by negative sides that form when aluminum replaces silicon. Additionally, sodium can form Si-O-Na bonds that break the silica network which leads to a more basic pH [100]. This turn to a basic pH could have some potential cytotoxic effect on cells.

Calcium is also a very important ion that needs to be released in order to achieve all the previous mentioned targets. In a previous study, a concern about reduction of calcium release from materials through time was reported [24]. However, in our study, release of calcium remained stable or presented an increase in most materials at two weeks observation period (apart from Fuji II LC). 

Conventional glass ionomer cements are considered to be biocompatible materials. Previous studies investigating the cytotoxicity of conventional and resin-modified glass ionomer cements have shown that resin-modified glass ionomer are more toxic than conventional glass ionomer cements [101,102,103]. Exposure of pulp cells to Fuji IX and Fuji II LC decreased the cell numbers in regard to controls [103]. However, only cells that were exposed to Fuji II LC presented morphological changes [103]. Additionally, in a previous study that examined the cytotoxicity of Fuji IX and Fuji II LC on Vero cells, none of the materials showed toxic effects after 24 and 48 h incubation periods [102]. However, after 75 h of incubation both materials presented significant lower cell viability values than the control group [102]. The use of bioactive materials continues to gain space against conventional restorative materials. In this study, Activa Bioactive Restorative revealed a good degree of biocompatibility to human gingival fibroblasts. However, the ions’ release was lower than that of conventional glass ionomer, and Activa exhibited a relatively remarkable release of organic eluates. These results are in accordance with a recently published study that examined cytotoxicity of a similar product Activa Bioactive Kids Restorative on DPCs [104]. Time of extraction was not found to be critical, but time of exposure was critical, since in both extraction times and 72 h exposure Ena HRi was more cytotoxic than Activa Bioactive Restorative. Especially at one week extract and 72 h measurement, Ena HRi decreased cell viability by 25% for 100% and 75% dilutions.

## 4. Materials and Methods

Ethical approval for this study was received from Ethics Committee of the Department of Dentistry, Aristotle University of Thessaloniki (approval number ID 29/21.11.2018).

### 4.1. Materials and Specimens Preparation for GC/MS and ICP–OES Analysis

Five commercially available restorative materials were employed for this study: Activa^TM^ Bioactive Restorative (Pulpdent, Watertown, MA, USA), ENA HRi (Micerium S.p.A., Avegno, Italy), Enamel plus HRi Biofunction (Micerium S.p.A., Avegno, Italy), Fuji II LC Capsule (GC America Inc., Alsip, IL, USA) and Fuji IX fast Capsule (GC America Inc., Alsip, IL, USA). Detailed information about composition of these materials according to manufacturers is shown in Table 8.

Teflon (PTFE) molds were filled with uncured material to produce disks with a diameter of 5 mm and thickness of 2 mm (according to ISO 4049:2019). The mold surfaces were overlaid with glass slides covered with a Mylar sheet to avoid air entrapping and adhesion of the final set material. The assembly was held together with spring clips and irradiated. The disks were polymerized with a curing LED light (Bluephase style, Ivoclar/Vivadent). The curing light was directly applied on the samples’ surface. All disks were cured for 20 s according to the manufacturer’s instructions. The power of the LED light ranged across all experiments in a range of 1100–1400mW/cm^2^, as tested by a special power meter. Disk specimens from Fuji IX fast with the same dimensions were left to set for 5 min in the molds. Thirty specimens of each material were prepared, and three identical series of experiments were conducted. Twenty specimens of each material were used for GC/MS analysis, while ten specimens of each material were used for ICP–OES analysis.

### 4.2. Monomer Elution Evaluation

Two series of fifty 8 mL amber glass vials were prepared, half of them filled with a solution of 1 mL of methanol (Methanol, HPLC gradient grade 99.9+%, CHEM-LAB) containing 0.1 mg caffeine (Caffeine 99%, Alfa Aesar) as internal standard, and half of them containing 1 mL of artificial saliva. The disks were detached from the molds and immediately immersed in methanol or artificial saliva solutions in the separate 8 mL amber glass vials. To simulate the condition changes of oral environment, artificial saliva was prepared by dissolving 0.40 g NaCl, 0.40 g KCl, 0.80 g CaCl_2_·2H_2_O, 1.00 g CH_4_N_2_O, 0.78 g NaH_2_PO_4_ and 0.005 g Na_2_S⋅9H_2_O in 1L of water for injection. Sodium chloride (NaCl) was provided by Centralchem, potassium chloride (KCl) from Chem-Lab NV, urea (CH_4_N_2_O) from Honeywell-Fluka, while calcium chloride dehydrate (CaCl_2_·2H_2_O), sodium phosphate monobasic (NaH_2_PO_4_) and sodium sulfide nonahydrate (Na_2_S⋅9H_2_O) were all purchased from Sigma-Aldrich. The 8 mL amber glass vials were sealed with polypropylene caps internally coated with PTFE septa to prevent evaporation. One set was kept in an incubator at 37 °C for 24 h, while the other series for one month. 

### 4.3. Specimens in Methanol

After 24 h and one month aging, 1 mL of each methanol solution was transferred to separate GC vials and injected into the gas chromatograph.

#### 4.3.1. Specimens in Artificial Saliva

After 24 h and one month aging, the specimens were removed from the amber glass vials and 1 mL of dichloromethane (Dichloromethane, GC gradient grade ≥99.9%, Sigma Aldrich) containing 0.1 mg (Caffeine 99%, Alfa Aesar) as internal standard was added into the artificial saliva solutions. The mixture was agitated for 1 min and left until complete phase separation. The organic layer was isolated, dried with anhydrous CaCl_2_, and extracts were filtered through PTFE filter discs (0.45 μm). The extracts were transferred to separate GC vials and injected into the gas chromatograph.

#### 4.3.2. Separation by Gas Chromatography and Mass Spectrometric Detection

The analyses were performed by using a gas chromatography/mass spectrometry instrument (GC/MS Clarus 500, Perkin Elmer, Shelton, Connecticut, USA) supported by a suitable software (Perkin Elmer, TurboMass version 5.4.2). The GC was equipped with an autosampler and a DB-5-MS capillary column (30 m, 0.25 mm id., 0.25 μm film, Agilent, Santa Clara, California, USA). The injector (split 1:20) was held at 250 °C. The initial temperature of the oven was held at 50 °C for 2 min and then increased at the rate of 10 °C/min to 300 °C and remained constant for 5 min. Helium 5.0 was used as the carrier gas with a constant flow rate of 1 mL/min. The transfer line from GC to MS was set to 310 °C. The mass spectrometer was operated in electron ionization mode (E.I.), while ion source was operated at 220 °C. Only positive ions were scanned. The syringe was rinsed 2 times before and after injection.

Identification and quantification of the analytes were performed by using mass spectrometer in full scan mode scanning from 50 to 450 *m*/*z* at a rate of 0.2 scan per second. NIST library (National Institute of Science and Technology, Gaithersburg, MD, USA), retention time and literature data were used for the identification of the different compounds. Caffeine was used as internal standard and analyzed at the m/z ratio of 194. For quantification, the respective peak of each compound was normalized on the caffeine peak. Reagent blank samples, containing just caffeine dissolved in methanol, were also analyzed. Methanol was injected between each of the samples to check if there were any carry-over effects during analysis. 

### 4.4. Ion Release

Specimens were immersed in 5 mL deionized water in separate amber glass vials. The vials were sealed with polypropylene caps internally coated with PTFE septa to prevent evaporation and kept in an incubator at 37 °C for 1 week. Afterwards the solutions were removed, and specimens were placed in 5 mL of fresh deionized water and stored for a further period of one week. Before the analysis, the solution samples were acidified with 10 mL of 10% HNO_3_ solution and passed through standard filter paper. Si, Al, Ca, P, Na and Ba ion concentrations measured using inductively coupled plasma–optical emission spectroscopy (ICP–OES, Optima 2100DV, Perkin Elmer). The optimum instrumental conditions were set as: 0.8 L min^−1^ nebulizer argon flow rate, 1300 W incident power and 1.5 mL min^−1^ sample flow rate. Specific emission lines were selected to identify each element as shown in Table 9. Three readings per element were conducted during sample running. Each sample was prepared and analyzed three times, and an average value per element was taken to obtain accurate results. Deionized water was also measured as blank solution for the baseline correction. Calibration curves for each of the examined ion were prepared using different dilutions of a multielement standard solution (Multielement standard solution for ICP, TraceCERT, SIGMA-ALDRICH).

### 4.5. Cell Viability Experiment

#### 4.5.1. Restorative Materials Extracts

The cytotoxicity of Fuji II LC and Fuji IX on various cell types have been tested previously [101,102,103,105,106,107]. Only Activa Bioactive restorative and Ena HRi effects on cell survival were investigated. The reason why only these two materials were selected for cytotoxicity test was their highest number of the organic eluates detected from these two materials after the GC-MS analysis, thus rendering them potential of inducing cytotoxic effects. Additionally, one of the purposes of our study was to compare the effect on cell survival of a conventional and a bioactive resin-based material. Thus, in the present study, we assessed the cytotoxic effects of Activa^TM^ Bioactive Restorative (Pulpdent, Watertown, MA, USA) and ENA HRi (Micerium S.p.A., Avegno, Italy). Activa Bioactive Restorative is a newly introduced material that is supposed to have bioactive abilities, while Ena is a “conventional” resin-based restorative material. The difference on materials’ synthesis was one additional reason to study the cytotoxic effects of these materials. Teflon molds were filled with uncured material to produce disks of 5 mm diameter and 2 mm thickness (according to ISO 4049:2019). The disks were polymerized with a curing LED light (Bluephase style, Ivoclar/Vivadent). The curing light was directly applied on the samples’ surface. All disks were cured for 20 s according to the manufacturer’s instructions. The power of the LED light ranged across all experiments in a range of 1100–1400mW/cm^2^, as measured by a special power meter. Fifteen specimens of each material were prepared. They were sterilized under ultraviolet rays for 15 min and stored in an incubator at 37°C for 48 h to set. Five milliliters of culture medium (Dulbecco modified Eagle medium [DMEM]; Life Technologies, Grand Island, NY) was added in the specimens. The specimens were incubated for 24 h and 1 week to extract the eluates (the total surface to volume ratio was 3 cm^2^/mL for polymers with diameter >0.5 mm was set according to ISO 10993-12). The extracts were sterile filtered with 0.22 μm filters. 

#### 4.5.2. Cell Culture

Human gingival fibroblasts (HGFs) were obtained from healthy marginal gingival tissue received from the palatal area of molars. The tissues were immediately placed in culture medium (Dulbecco modified Eagle medium [DMEM]; Life Technologies, Grand Island, NY) for at least 1 h, rinsed three times in phosphate-buffered saline solution (PBS, EuroCloneSpA), minced into small tissue pieces, and cultured in 75cm^2^ culture flasks at 37°C in a humidified atmosphere with 5% CO_2_. DMEM supplemented with 10% fetal bovine serum and antibiotics (100 IU/mL penicillin and 100 mg/mL streptomycin) was used as culture medium (20 mL culture medium per flask). Cell viability was assessed with the trypan blue (Sigma-Aldrich, St Louis, MO, US) exclusion method in a hemocytometer Newbauer (Improved Bright-line; HBG, Giessen, Hessen, Germany). Adherent cells in a logarithmic growth phase were seeded (100 μL/well) in 96-well flat-bottom microtiter plates (Corning Costar, New York, NY, US) at a concentration of 3000 cells/well. The extracts were added to the cells at 100%, 75%, 50% and 25% dilution using culture medium as the dilution material. A total of 200 μL DMEM was added to the cells that served as negative controls.

#### 4.5.3. Cell Viability

Cell viability was estimated at 24 h and 72 h of exposure, with a modified staining sulforhodamine B assay (Sigma-Aldrich) in reference to controls as described by Papazisis et al. [108]. The sulforhodamine B (SRB) assay is used for cell density determination and is based on the measurement of cellular protein content. This method relies on the property of SRB, which binds stoichiometrically to proteins under mild acidic conditions and then can be extracted using basic conditions. Additionally, the amount of bounded dye can be used as a proxy for cell mass, which then can be extrapolated in order to measure cell proliferation [109]. Viability was estimated by fraction unaffected, which is the proportion of cells that were not affected from the agent and derived from the following equation: fraction unaffected = ODx/ODc. ODx represented the test optical density, and ODc represented the control optical density. The mean of the 6 measurements for each concentration tested, the standard deviation, and the coefficient of variation were calculated. Dose-response curves were plotted for each agent. 

### 4.6. Statistical Analysis

The results are presented as median values with associated interquartile range. Statistical analysis was performed using IBM SPSS software, version 25, using one-way ANOVA, followed by Tukey’s posthoc test and Fisher’s exact test with an assumed level of significance *p* < 0.05.

## 5. Conclusions

No organic substances were detected from the glass ionomer cement Fuji IX fast. More organic species of higher concentrations were detected in methanolic extracts than in artificial saliva extracts, which may imply the protective action of saliva. Elution of organic substances depended on material’s composition, the nature of the solvent and the storage time. Τhe ions silicon, aluminum, calcium, sodium and barium were detected from all materials, while phosphorus was not detected from any of the materials. Ions, apart from calcium, presented a time-dependent decrease. As far as cytotoxic effects concerns, the newly introduced bioactive restorative was found to be more biocompatible compared to the conventional resin-based restorative.

## Figures and Tables

**Table 1:** Eluted substances arranged by increasing retention time, with abbreviation, molecular formula, compound name, molecular weight, characteristic ions and chemical structure.

Eluate	Retention Time (RT)	Abbreviation	Molecular Formula	Compound Name	Molecular Weight	Characteristic ions, *m*/*z*	Chemical Structure
1	7.08	MS	C_9_H_10_	α-Methylstyrene	118	118, 103, 78, 115	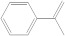
2	7.55	HEMA	C_6_H_10_O_3_	2-Hydroxyethyl methacrylate	130	69, 87	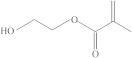
3	8.51	ACP	C_8_H_8_O	Acetophenone	120	105, 77, 120, 51	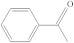
4	8.82	MOPA	C_9_H_10_O_3_	Methoxyphenyl acetic acid	166	121, 77, 51, 78	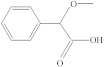
5	8.87	MCE	C_10_H_14_O	Methyl cumyl ether	150	135, 91, 77, 73, 136	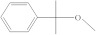
6	10.71	MeHQ	C_7_H_8_O_2_	Mequinol	124	109, 124, 81	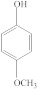
7	12.14	CQ	C_10_H_14_O_2_	Camphorquinone	166	95, 69, 83	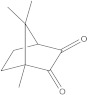
8	14.77	BHT	C_15_H_24_O	Butylated hydroxytoluene	220	205, 220, 57	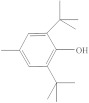
9	15.12	1,4-BDDMA	C_12_H_18_O_4_	1,4-Butylene glycol dimethacrylate	226	69, 55, 112	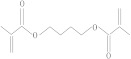
10	17.23	DMABEE	C_11_H_15_O_2_N	Ethyl 4-(dimethylamino) benzoate	193	148, 193, 164	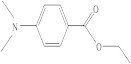
11	18.69	IS	C_8_H_10_O_2_N_4_	Caffeine	194	194, 109	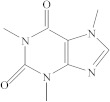
12	18.91	DBP	C_16_H_22_O_4_	Dibutyl phtalate	278	149, 150, 57	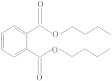
13	20.34	TMPTMA	C_18_H_26_O_6_	Trimethylolpropane trimethacrylate	338	69, 253	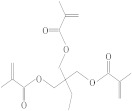
14	21.92	BPA	C_15_H_16_O_2_	Bisphenol A	228	213, 119	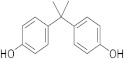
15	22.79	TPSb	C_18_H_15_Sb	Triphenylstibine	352	198, 154, 200	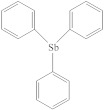

**Table 2:** Analytes detected in artificial saliva of the five investigated materials for both observation periods (24 h and 1 month). The numbers correspond to the eluates reported in Table 1.

	Organic Substances Detected in Artificial Saliva																													
	1		2		3		4		5		6		7		8		9		10		11		12		13		14		15	
	24 h	1 m	24 h	1 m	24 h	1 m	24 h	1 m	24 h	1 m	24 h	1 m	24 h	1 m	24 h	1 m	24 h	1 m	24 h	1 m	24 h	1 m	24 h	1 m	24 h	1 m	24 h	1 m	24 h	1 m
*Activa Bioactive Restorative*			√		√		√	√					√				√				√	√	√	√	√					
*Ena HRi*													√	√							√	√	√	√						
*Enamel Plus HRiBioFunction*													√								√	√	√							
*GC Fuji II LC*			√	√									√								√	√								
*GC Fuji IX*																					√	√								

**Table 3:** Analytes detected in methanol of the five investigated materials for both observation periods (24 h and 1 month). The numbers correspond to the eluates reported in Table 1.

	Organic Substances Detected in Methanol																													
	1		2		3		4		5		6		7		8		9		10		11		12		13		14		15	
	24 h	1 m	24 h	1 m	24 h	1 m	24 h	1 m	24 h	1 m	24 h	1 m	24 h	1 m	24 h	1 m	24 h	1 m	24 h	1 m	24 h	1 m	24 h	1 m	24 h	1 m	24 h	1 m	24 h	1 m
*Activa Bioactive Restorative*	√	√	√	√	√	√	√	√	√	√			√	√			√	√	√	√	√	√			√	√				
*Ena HRi*			√	√									√	√	√	√	√	√	√	√	√	√					√	√	√	√
*Enamel Plus HRi BioFunction*			√	√								√	√	√	√	√	√	√	√	√	√	√								
*GC Fuji II LC*			√	√									√	√							√	√								
*GC Fuji IX*																					√	√								

**Table 4:** Median values of relative amounts (%CF) for eluates measured in artificial saliva extracts for both observation periods (24 h and 1 month).

	Activa Bioactive Restorative		Ena HRi		Enamel Plus Hri BioFunction		GC Fuji II LC	
	24 h	1 m	24 h	1 m	24 h	1 m	24 h	1 m
*MS*								
*ACP*	4.49 (9.32)							
*MCE*								
*BHT*								
*BPA*								
*1,4-BDDMA*	5.24 (100.11)							
*CQ*	0.24 (9.91)		19.45 (94.94) ^*^	1,24 (31.86) ^**^	1.30 (3.15) ^*^		0.21 (1.60)	
*DMABEE*								
*DBP*	69.96 (13.05) ^*^	32.51 (76.07) ^**^	131.76 (50.7) ^*^	43.79 (223.36) ^**^	25.80 (30.66) ^*^			
*HEMA*	8.85 (92.93) ^*^						1.93 (0.62) ^*^	4.80 (6.22) ^**^
*MeHQ*								
*MOPA*	160.60 (27.61)	149.38 (181.21)						
*TMPTMA*	0.40 (0.15)							
*TPSb*								

^*^ Indicates significant difference among the materials, ^**^ indicates significant difference according to observation periods.

**Table 5:** Median values of relative amounts (%CF) for eluates measured in methanol extracts for both observation periods (24 h and 1 month).

	Activa Bioactive Restorative		Ena HRi		Enamel Plus Hri BioFunction		GC Fuji II LC	
	24 h	1 m	24 h	1 m	24 h	1 m	24 h	1 m
*MS*	43.77 (20.25)	81.28 (27.59) ^**^						
*ACP*	59.71 (23.96)	95.95 (34.29)						
*MCE*	110.00 (49.18)	164.00 (81.43)						
*BHT*			3.91 (3.38) ^*^	12.58 (7.05) ^*^^,^**	11.58 (5.46) ^*^	29.60 (58.57) ^*,**^		
*BPA*			0.91 (0.74)	0.72 (0.76)				
*1,4-BDDMA*	554.23 (217.34) ^*^	369.27 (121.63) ^*^	603.47 (295.91) ^*^	636.15 (311.61) ^*^	137.43 (107.92) ^*^	104.34 (158.85) ^*^		
*CQ*	12.08 (2.02) ^*^	28.19 (8.26) ^*,**^	5.20 (2.75) ^*^	8.70 (5.34) ^*^	1.19 (0.97) ^*^	3.16 (4.79) ^*,**^	10.85 (1.83) ^*^	24.22 (14.47) ^*,**^
*DMABEE*	33.97 (19.55) ^*^	99.02 (38.73) ^*,**^	5.74 (4.87) ^*^	13.90 (15.13) ^*,**^	4.62 (3.69) ^*^	18.63 (10.72) ^*,**^		
*DBP*								
*HEMA*	694.44 (229.71) ^*^	854.14 (158.63) ^*^	306.72 (235.00) ^*^	363.76 (237.71) ^*^	178.95 (116.71) ^*^	169.28 (141.01) ^*^	25.76 (9.16) ^*^	20.75 (4.69) ^*^
*MeHQ*						7.18 (8.17)		
*MOPA*	890.78 (576.86)	1372.61 (430.58)						
*TMPTMA*	40.11 (24.31)	64.40 (21.33)						
*TPSb*			0.47 (0.69)	4.39 (1.88) ^**^				

^*^ Indicates significant difference among the materials, ^**^ indicates significant difference according to observation periods.

**Table 6:** Ions released in ppm for both observation period (1 and 2 weeks).

	Si		Al		Ca		Na		Ba	
	1 w	2 w	1 w	2 w	1 w	2 w	1 w	2 w	1 w	2 w
*Activa Bioactive Restorative*	1.22 ± 0.29	0 *,**	1.08 ± 0.68	0.46 ± 0.03 ^*,**^	0.7 ± 0.21 ^**^	0.69 ± 0.08	2.71 ± 0.14	2.21 ± 0.04	0.16 ± 0.03	0.06 ± 0.02
*Ena HRi*	0.8 ± 0.2	0 ^*,**^	0.87 ± 0.87	0.85 ± 0.17 ^*^	0.19 ± 0.05	0.6 ± 0.2 ^**^	2.38 ± 0.34	2.09 ± 0.02	0.97 ± 0.02 ^*,**^	0.06 ± 0.008 ^**^
*Enamel Plus HRi Biofunction*	0.27 ± 0.08 ^*^	0 ^*^	0.31 ± 0.06 ^*^	0.81 ± 0.54 ^*,**^	0.29 ± 0.02	0.78 ± 0.09 ^**^	2.2 ± 0.11	0 ^**^	0.07 ± 0.01	0.06 ± 0.01
*Fuji II LC*	0.91 ± 0.59	0.41 ± 0.3 ^*,**^	2.34 ± 0.3 ^*^	0.84 ± 0.44 ^*,**^	0.75 ± 0.29 ^**^	0.39 ± 0.42 ^*,**^	3.65 ± 0.26	2.39 ± 0.12	0.11 ± 0.01	0.09 ± 0.01
*Fuji IX*	11.17 ± 2.12 ^*,**^	2.46 ± 0.8 ^*,**^	5.73 ± 0.81 ^*,**^	1.18 ± 0.24 ^*,**^	0.25 ± 0.05	0.7 ± 0.39 ^**^	7.32 ± 0.74 ^*,**^	3.22 ± 0.2 ^**^	0.11 ± 0.03	0.09 ± 0.008

^*^ Indicates significant difference among the materials, ^**^ indicates significant difference according to observation periods.

**Table 7:** Mean values and standard deviations for cell viability test.

24 h Extract/24 h Measurement				
	100%	75%	50%	25%
*Ena HRi*	101 ± 9.54	102 ± 6.56	97 ± 3.46	101.33 ± 2.89
*Activa Bioactive Restorative*	109 ± 5.13	108 ± 3.61	102 ± 4	101.7 ± 2.08
24 h extract/72 h measurement				
	100%	75%	50%	25%
*Ena HRi*	97 ± 13	96.7 ± 9.02	95 ± 4.51	99 ± 3
*Activa Bioactive Restorative*	116 ± 13.87	113 ± 11.93	108 ± 6.65	105 ± 4.73
1 week extract/24 h measurement				
	100%	75%	50%	25%
*Ena HRi*	115 ± 30.75	113 ± 25.24	108 ± 20.22	111 ± 22.81
*Activa Bioactive Restorative*	108 ± 7.81	108 ± 7.5	116 ± 15.8	107 ± 9.64
1 week extract/72 h measurement				
	100%	75%	50%	25%
*Ena HRi*	75 ± 26.1 ^*^	75 ± 23.46 ^*^	83 ± 16.01	89.7 ± 34.67
*Activa Bioactive Restorative*	99 ± 10.58	101 ± 6.65	98 ± 8.5	99.7 ± 3.79

^*^ Indicates significant difference among the materials.

**Table 8:** Material composition according to Material Safety Data Sheets.

Material	Manufacturer	Composition according to MSDS
Activa Bioactive Restorative	Pulpdent, Watertown, MA, USA	Blend of diurethane and other methacrylates with modified polyacrylic acid (44.6%) Silica, amorphous (6.7%) Sodium fluoride (0.75%)
Ena HRi	Micerium S.p.A., Avegno, Italy	1,4-Butanediol dimethacrylate Urethane dimethacrylate Bis-GMA Tetramethylenedimethacrylate (2.5–10%) ^*^ CONTENT OF THE FILLERS: 74% by weight (60% by volume); particle size of highly dispersed silicone dioxide is 0.005–0.05 μm, glass fillers have a particle size of 0.2–3.0 μm.
Enamel Plus HRi BioFunction	Micerium S.p.A., Avegno, Italy	Tricyclodecanedimethanoldimethacrylate (10–25%) 2-propenoic acid, 2-methyl-, 3- (trimethoxysilyl) propyl ester, reaction products with Silicon dioxide (2.5–10%) ^*^ TOTAL CONTENT OF THE FILLERS: 74% by weight (60% by volume); particle size of highly dispersed silicone dioxide is 0.005–0.05 μm, glass fillers have a particle size of 0.2–3.0 μm
GC Fuji II LC	GC America Inc., Alsip, IL, USA	2-hydroxyethyl methacrylate (HEMA) (25–50%) Polybasic carboxylic acid (5–10%) Urethane dimethacrylate (UDMA) (1–5%) Dimethacrylate (1–5%)
GC Fuji IX	GC America Inc., Alsip, IL, USA	Polybasic carboxylic acid (5–10%)

^*^ Additional composition information from materials leaflets.

**Table 9:** ICP–OES emission lines selected for ions’ identification.

Ion	Wavelength (nm)
Si	251.611
Al	396.153
Ca	393.366
P	213.617
Na Ba	588.995 455.403

## Data Availability

The datasets used and/or analyzed during the current study are available from the corresponding author on reasonable request.

## Sample Availability

The samples of the compounds are not available.
